# Eeyarestatin 1 Interferes with Both Retrograde and Anterograde Intracellular Trafficking Pathways

**DOI:** 10.1371/journal.pone.0022713

**Published:** 2011-07-25

**Authors:** Mina-Olga Aletrari, Craig McKibbin, Helen Williams, Vidya Pawar, Paola Pietroni, J. Michael Lord, Sabine L. Flitsch, Roger Whitehead, Eileithyia Swanton, Stephen High, Robert A. Spooner

**Affiliations:** 1 School of Life Sciences, University of Warwick, Coventry, United Kingdom; 2 Faculty of Life Sciences, University of Manchester, Manchester, United Kingdom; 3 School of Chemistry, University of Manchester, Manchester, United Kingdom; 4 Manchester Interdisciplinary Biocentre, University of Manchester, Manchester, United Kingdom; Cornell University, United States of America

## Abstract

**Background:**

The small molecule Eeyarestatin I (ESI) inhibits the endoplasmic reticulum (ER)-cytosol dislocation and subsequent degradation of ERAD (ER associated protein degradation) substrates. Toxins such as ricin and Shiga/Shiga-like toxins (SLTx) are endocytosed and trafficked to the ER. Their catalytic subunits are thought to utilise ERAD-like mechanisms to dislocate from the ER into the cytosol, where a proportion uncouples from the ERAD process, recovers a catalytic conformation and destroys their cellular targets. We therefore investigated ESI as a potential inhibitor of toxin dislocation.

**Methodology and Principal Findings:**

Using cytotoxicity measurements, we found no role for ES_I_ as an inhibitor of toxin dislocation from the ER, but instead found that for SLTx, ESI treatment of cells was protective by reducing the rate of toxin delivery to the ER. Microscopy of the trafficking of labelled SLTx and its B chain (lacking the toxic A chain) showed a delay in its accumulation at a peri-nuclear location, confirmed to be the Golgi by examination of SLTx B chain metabolically labelled in the *trans*-Golgi cisternae. The drug also reduced the rate of endosomal trafficking of diphtheria toxin, which enters the cytosol from acidified endosomes, and delayed the Golgi-specific glycan modifications and eventual plasma membrane appearance of tsO45 VSV-G protein, a classical marker for anterograde trafficking.

**Conclusions and Significance:**

ESI acts on one or more components that function during vesicular transport, whilst at least one retrograde trafficking pathway, that of ricin, remains unperturbed.

## Introduction

**Figure 1:**
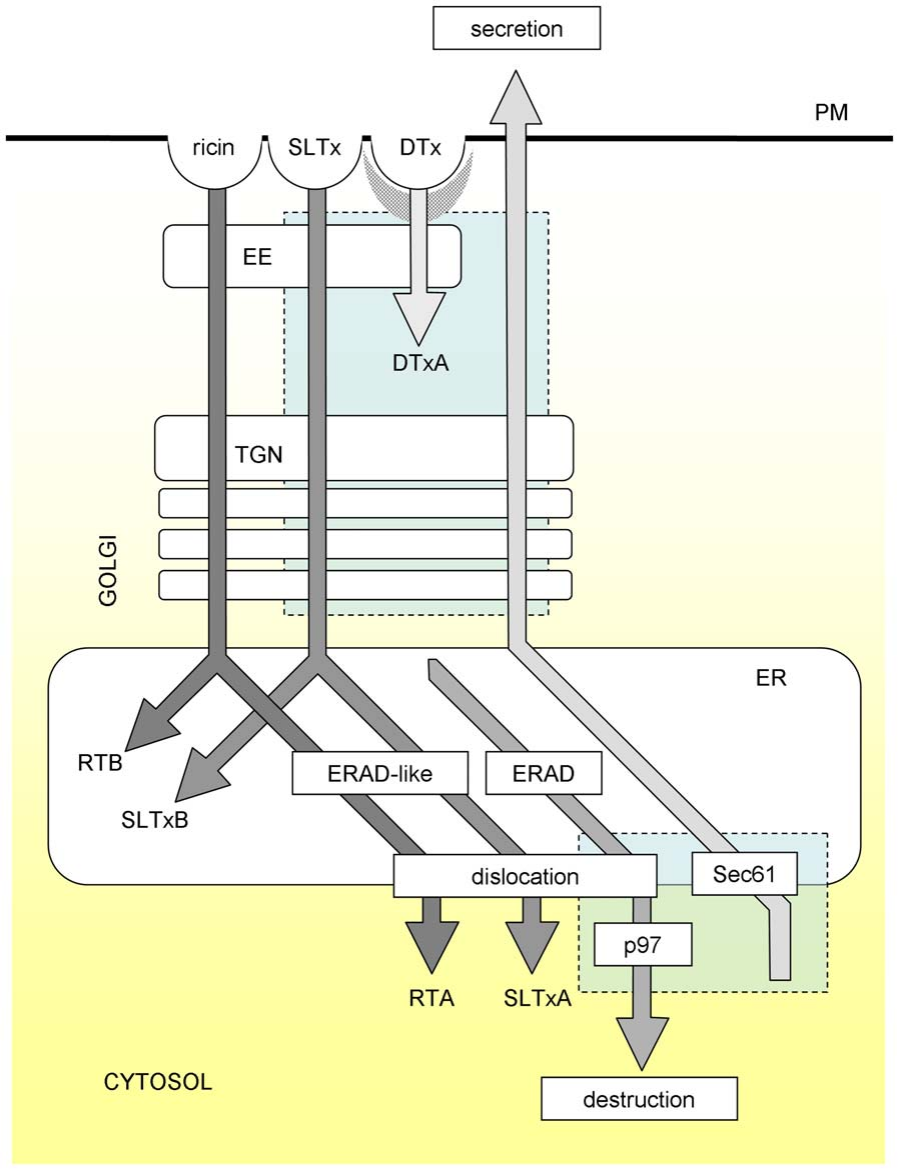
Vesicular trafficking schemes. Following clathrin-independent endocytosis, the cytotoxic fractions of ricin and Shiga –like toxin (SLTx) traffic to the ER *via* early endosmes (EE), the *trans*-Golgi network (TGN) and the Golgi apparatus to the endoplasmic reticulum (ER), where the ricin subunits RTA and RTB are reductively separated. It is assumed that SLTxA chain is also separated from its B chain pentamer (SLTxB) in the ER. The toxic RTA and SLTxA chains are thought to co-opt ERAD mechanisms to dislocate from the ER to the cytosol, where they are refolded to recover toxic catalytic activity (ribosome modification). Diphtheria toxin (DTx) is endocytosed in a clathrin-dependent manner (hatched area) and enters the cytosol from acidified endosomes. In the cytosol it is reduced, releasing the A chain (DTxA) which inactivates elongation factor 2. Blue boxes, areas/processes affected by ES_I_: lower box, previously identified targets; upper box, this study. PM, plasma membrane.

The plant toxin ricin and the bacterial Shiga and Shiga-like toxins (SLTx) bind their receptors (glycoproteins/glycolipids bearing exposed β1→4 linked galactose residues and the sphingolipid Gb3 respectively) at the mammalian cell surface and, after internalization by endocytosis, traffic in vesicular carriers to the endoplasmic reticulum (ER) [1] (Figure 1). Here, the toxic A chain (RTA) of ricin is reductively separated from its receptor-binding B chain (RTB) [2], [3] and then unfolds and partially inserts into the ER membrane [4]. This change in structure is thought to mimic a misfolded membrane-associated protein, allowing RTA to co-opt components of the ER-associated protein degradation (ERAD) pathways that remove misfolded proteins and orphan subunits from the ER in order to maintain ER homeostasis [5], [6], [7].

Typical soluble ERAD substrates are unfolded in the ER lumen and then extracted (dislocated) to the cytosol. During dislocation, substrates are usually polyubiquitylated on internal lysine residues by membrane integral E3 ubiquitin ligases such as HRD1. This process provides tags for the cytosolic AAA (ATPase associated with various cellular activities)-ATPase extraction motor p97/VCP [8], [9], [10] and subsequently, after limited de-ubiquitylation [11], [12], [13] for the proteasome [14], which is the terminal destination for ERAD substrates. RTA can be co-immunoprecipitated with the Sec61 translocon [15], which is a putative component of the dislocation machinery [16], and with EDEM, an ERAD-associated mannosidase [17]. Furthermore, RTA dislocation and cytotoxicity are sensitive to manipulation of expression levels of SEL1L [18], a regulator of the HRD complex. All these observations are consistent with use by RTA of endogenous ERAD components. Post-dislocation, cytosolic triage by molecular chaperones permits folding of a proportion of cytosolic RTA to an active conformation and therefore at least a fraction of dislocated RTA uncouples from the final ERAD step of proteasomal degradation [19]. Refolded cytosolic RTA then depurinates the large ribosomal subunit at one specific site [20], resulting in cessation of protein synthesis and ultimately in cell death. The RTA dislocation process in mammalian cells remains otherwise unmapped. However, when expressed in the ER of the yeast *Saccharomyces cerevisiae*, RTA also utilizes components of the yeast equivalent of the mammalian HRD1 complex (the Hrd1p complex) for dislocation [21]. SLTx A chain (SLTxA) has identical catalytic activity to RTA [22], and the holotoxin traffics to the ER in a superficially similar manner to ricin [1] (Figure 1). It is widely assumed that SLTxA also utilizes ERAD components for dislocation to the cytosol. Little is known about the dislocation process for SLTxA, other than interactions with ER chaperones and Sec61 prior to dislocation [23], [24].

Mammalian major histocompatibility complex (MHC) Class I heavy chains are normally expressed as type I membrane proteins in the mammalian endoplasmic reticulum (ER), where they are chaperoned until assembly with their partner β-microglobulin chains [25], [26]. Each complex then binds a peptide derived from proteins degraded in the cytosol that enters the ER lumen via transporters associated with antigen processing (TAPs). The assembled complex is trafficked via secretory vesicle carriers to the plasma membrane, allowing peptide display to cells of the immune system [27]. The cytomegalovirus immunoevasin protein US11 down-regulates the expression of assembled complexes by stimulating dislocation of folded MHC Class I molecules from the ER to the cytosol. Here they are deglycosylated and then degraded by the proteasome [28]. US11 interacts with numerous components [29], [30] of the ER membrane integral HRD1 ubiquitylation complex which is responsible for polyubiquitylating the MHC Class I heavy chains during dislocation [31], [32]. This HRD complex is fully functional in the absence of US11, dislocating unassembled MHC Class I heavy chains [33] as well as other ERAD substrates [5], [6]. The small molecule eeyarestatin I (ES_I_) was identified in a high throughput screen for increased fluorescence of mammalian cells expressing both an enhanced green fluorescent protein (GFP)-Class I MHC heavy chain fusion and the US11 gene product. ES_I_ was subsequently shown to also inhibit ERAD of orphan T cell receptor α (TCRα) subunits in the absence of US11 [34]. The mechanism of ES_I_ action is not yet clear and the initial suggestion that it interfered with pre-dislocation steps [34] was followed by evidence of an effect on a post-dislocation step, namely p97-associated de-ubiquitylation [35], [36], [37], a perturbation of co-translational translocation at the ER, most likely through inhibition of Sec61 functions [38], and formation of cross-linked adducts in the ER lumen between protein disulphide isomerase (PDI) and an ERAD substrate [39].

The degradation of ERAD substrates normally requires both ER dislocation and p97 activities, and since ES_I_ has been reported to perturb both processes [34], [35], [38], we investigated its effects on the cytotoxicity of ER-trafficking toxins. We report here that the later stages of the cytotoxic process for two toxins, ricin and SLTx, are unaffected by ES_I_ treatment (see Figure 1). This suggests that in both cases the dislocation of their respective toxic A chains across the ER membrane, and their subsequent delivery into the cytosol, is unperturbed by the compound. However, we find that SLTx toxicity is specifically reduced by ES_I_ treatment. We show that this effect results from interference with a much earlier stage of the cytotoxic process, resulting in a delay in the delivery of the holotoxin to the ER. Further studies revealed that ES_I_ can interfere with both the anterograde and retrograde transport of vesicular cargoes through the endomembrane system and we conclude that the ES_I_ is most likely capable of perturbing multiple cellular targets.

## Results

### ES_I_ treatment protects HeLa cells from challenge with SLTx but not ricin

**Figure 2:**
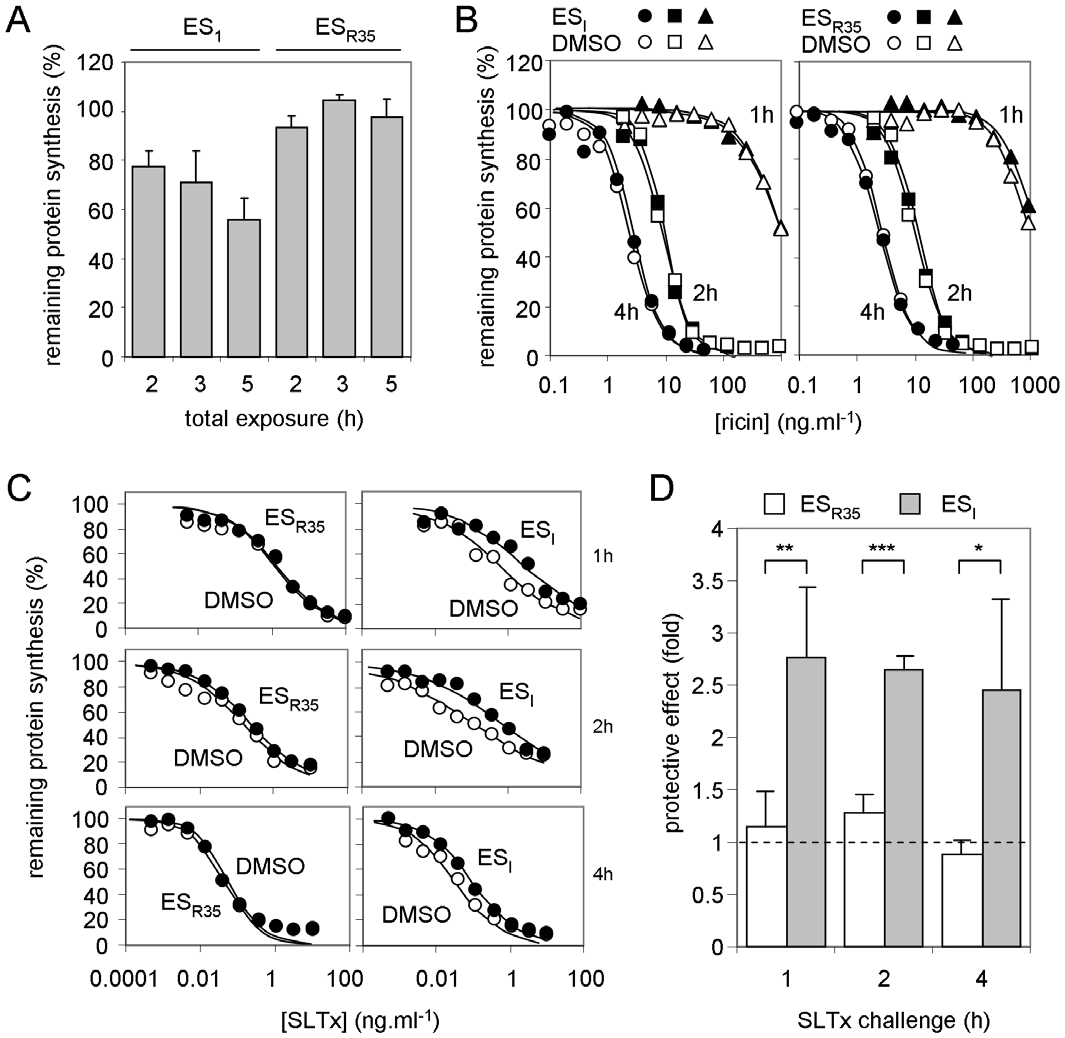
ES_I_ treatment protects HeLa cells from SLTx, but not from ricin. **A. ES_I_ treatment reduces cellular protein synthesis levels.** Cells were treated with ES_I_ or its inactive derivative ES_R35_ for 2, 3 or 5 h, and remaining protein synthesis ability was determined, shown relative to control cells treated with the vehicle DMSO. N = 3, bars +/− 1 S.D. **B. ES_I_ treatment has no effect on ricin intoxication.** Cells treated with DMSO (open symbols), ES_I_ (filled symbols, left-hand panel) or ES_R35_ (filled symbols, right hand panel) were challenged for 1 (circles), 2 (squares) or 4 h (triangles) with graded doses of ricin and remaining protein synthesis levels were determined. Typical single assays are shown. **C, D. ES_I_ treatment protects cells from SLTx challenge.** Cells treated with DMSO, ES_I_ or ES_R35_ were challenged with graded doses of SLTx for 1 (upper), 2 (middle) or 4 h (lower) and remaining levels of protein synthesis ability were determined. Typical single assays are shown in **C**, from which the IC_50_ (concentration of SLTx that reduced protein synthesis to half that of control non-toxin treated cells treated in parallel) values were determined, and means of 3 or 4 independent experiments (+/− 1 S.D.) are shown in **D**, expressed as fold protection (IC_50_ drug treated cells: IC_50_ DMSO treated cells). Protective effects from ES_I_ and ES_R35_ treated cells were compared using an unpaired t-test (1 h, p = 0.0048, t = 4.358, n = 4; 2 h, p<.0001, t = 12.28, n = 4; 4 h, p = 0.0377, t = 3.060, n = 3; *, significant: **, very significant: ***, extremely significant).

The toxic A chains of ricin and SLTx have identical RNA *N*-glycosidase activities, removing a single adenine residue from the large ribosomal subunit at the site of interaction with elongation factor complexes [20], [22], resulting in a cessation of protein synthesis. Cytotoxicity can therefore be determined by comparing the remaining levels of *de novo* protein synthesis capability in toxin-challenged cells to those in non-toxin treated cells by pulsing toxin-treated and control cells with radiolabelled amino-acids and measuring their incorporation into acid-precipitable material (proteins). HeLa cells were pretreated with ES_I_ or its inactive derivative ES_R35_ [38] for 1 h and then challenged for 1, 2 or 4 h with dilutions of ricin or SLTx in medium containing ES_I_ or ES_R35_ as appropriate, such that the compounds were present throughout the toxin challenge period (see Methods). Remaining protein synthesis at each toxin dilution was normalized to that of drug-treated, but non-toxin treated, controls. For each individual experiment a coeval control was performed, substituting DMSO (the vehicle in which ES_I_ and ES_R35_ were dissolved) for ES_I_ or ES_R35_, and here protein synthesis levels were normalized to DMSO-treated, but not toxin-treated, controls. ES_I_ treatment alone had some toxicity in this assay, since it reduced the protein synthesis ability of the cells (Figure 2A), but it had no obvious effect on the cytotoxicity of ricin (Figure 2B). In contrast, ES_I_ treatment protected cells ∼2.5 fold from challenge with SLTx (Figures 2C, D). ES_R35_ had little or no effect in any of these assays.

**Figure 3:**
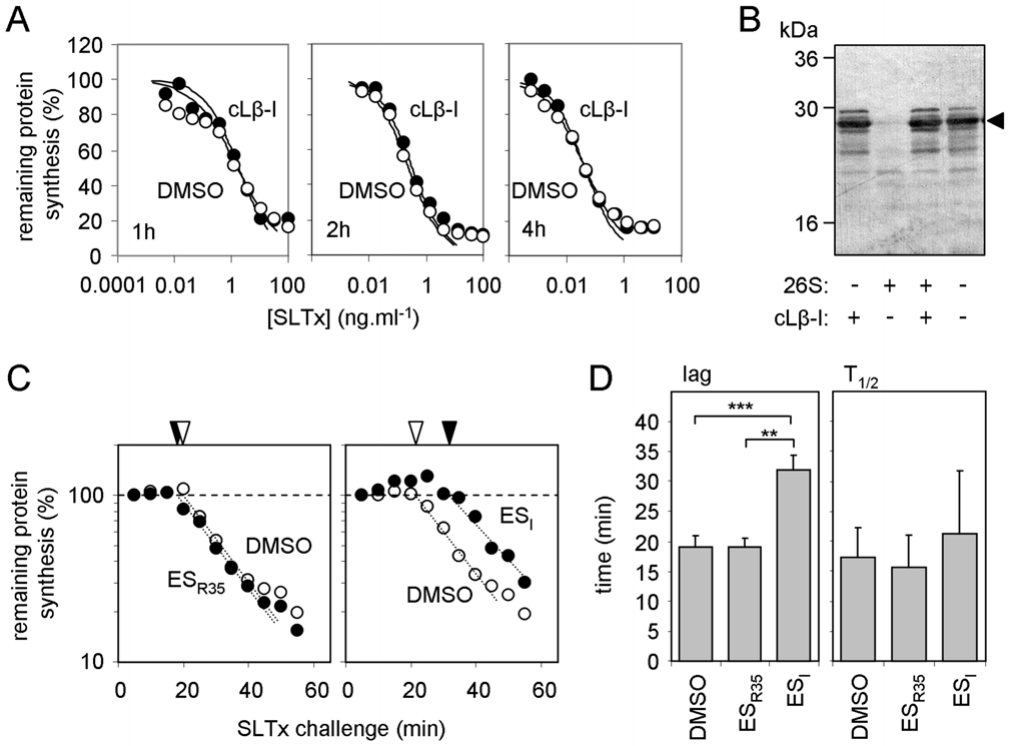
ES_I_ causes a delay in intoxication of HeLa cells with SLTx. **A, B. The UPS has little influence on SLTx cytotoxicity.** **A.** Cells treated with the proteasome inhibitor *clasto* Lactacystin β-lactone (cLβ-l) were challenged with SLTx for 1 (left), 2 (middle) or 4 h (right) and remaining levels of protein synthesis were determined. **B.** Efficacy of cLβ-l was confirmed *in vitro* by its ability to block degradation of casein (arrowhead) in the presence of mammalian 26S proteasomes. **C.** Cells treated with ES_R35_ (left hand panel) or ES_I_ (right hand panel) were challenged with a fixed dose (1 µg.ml^−1^) of SLTx for increasing periods and protein synthesis levels were determined. Exponential fits (dotted lines) were extrapolated to determine the lag phase of intoxication (white arrowhead, DMSO; black arrowhead, drug). Typical single assays are shown. **D.** Lag phases were determined as in **C**, and the half-lives of protein synthesis (T_1/2_) were determined from exponential fits in **C.** DMSO, n = 6, +/− 1.S.D., ES_I_ and ES_R35_, n = 3, +/− 1 S.D. Values were compared using unpaired t-tests. Lags: DMSO *versus* ES_R35_, no significant difference; DMSO versus ES_I_, p<0.0001, t = 8.347; ES_I_ versus ES_R35_ p = 0.0016, t = 7.658; **, very significant: ***, extremely significant. T_1/2_ values: no significant differences.

These results initially suggested a mechanistic difference in the ER to cytosol dislocation of RTA and SLTxA. ES_I_ perturbs the mammalian ubiquitin proteasome system (UPS) [35], so we examined the role of the proteasome in SLTx cytotoxicity to investigate whether this underlies its protective effect against SLTx but not ricin whose A chain dislocates independently of the UPS [21]. HeLa cells were challenged with SLTx in the presence of clasto-Lactacystin β-lactone (cLβ-l), an irreversible inhibitor of the three proteolytic activities of the proteasome [40]. However, despite a previous report that cLβ-l sensitizes Vero cells slightly to SLTx challenge [41], we saw no obvious effect in HeLa cells (Figure 3A). The effectiveness of cLβ-l was confirmed *in vitro*, by its inhibition of proteasomal degradation of casein (Figure 3B). Although the lack of obvious role for the proteasome in SLTx toxicity to HeLa cells may reflect a cell-type specific difference in the cytosolic fate of SLTxA, it should be noted that the previously reported effect of cLβ-l on SLTx cytotoxity was rather modest [41]. Our results suggest that the cytotoxicity of SLTxA is not strongly influenced by the UPS, and the protective effect of ES_I_ is most likely upstream of this process.

The mechanism of ES_I_ -dependent protection against SLTx was investigated further by examining the temporal profile of SLTx intoxication. HeLa cells were treated with a saturating dose of toxin (1 µg.ml^−1^) for increasing lengths of time and the remaining ability of the cells to synthesise proteins was measured. In such experiments, there is a distinct lag before the first measurable loss of protein synthesis ability, followed by a reduction in total cell population protein synthesis ability whose (log) slope is determined by the dislocation rate [42], [43]. If ES_I_ reduces SLTxA dislocation, this would be measurable by a reduction in the log slope of the exponential phase, but the pre-cytosolic phase (lag) should be unaltered. We found little or no difference in the half-time (T_1/2_, the time taken to reduce remaining protein synthesis ability by 50%) of the exponential phase. Strikingly, however, we measured instead a distinct intoxication lag phase increase of ∼20 minutes for ES_I_ treatment, whilst ES_R35_ had no measurable effects in these experiments (Figure 3C, D). This observation suggests that the step affected by ES_I_ most likely involves the delivery of the toxin to the ER, and is hence upstream of dislocation (see Figure 1).

### ES_I_ treatment perturbs SLTx trafficking

**Figure 4:**
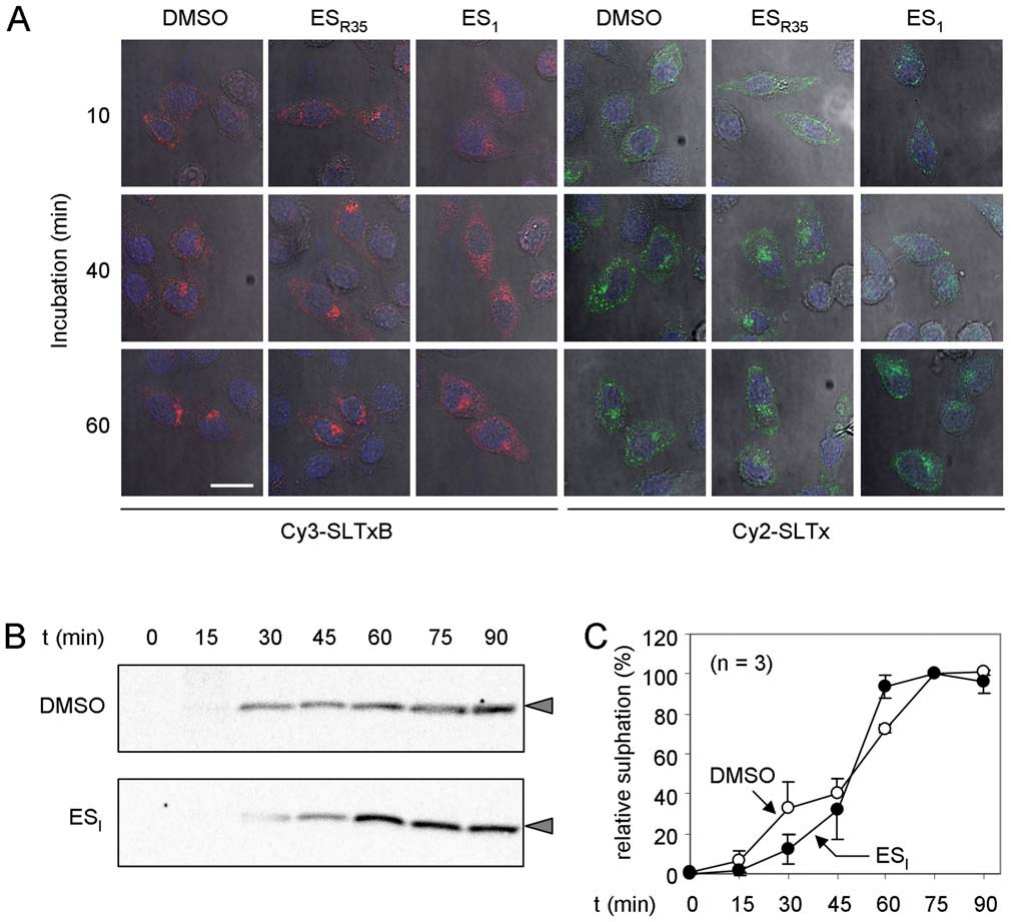
ES_I_ treatment interferes with retrograde trafficking of SLTx. **A.**Cells were treated with DMSO, ES_I_ or ES_R35_ and then incubated for increasing periods with Cy3-labelled SLTxB (red) or Cy2-labelled SLTx (green), after which they were fixed and examined by confocal microscopy. Blue, DAPI-stained nuclei, white scale bar, 25 µm. **B.** Cells starved of sulphate were incubated with [^35^S]O_4,_ treated with DMSO or ES_I_ and then incubated for increasing periods with a modified recombinant SLTxB pentamer, whose monomers bear a sulphation motif that is sulphated in the *trans*-Golgi cisternae. SLTxB-sulf (arrowheads) was immunoprecipitated from detergent lysates of the cells and revealed after SDS-PAGE by fluorography and autoradiography. **C.** Relative sulphation of SLTxB-sulf, determined from experiments performed in **B**, n = 3, +/− 1 S.D.

To examine whether ESI treatment delays delivery of SLTx to the ER, we applied fluorescently-labeled SLTx B chain pentamer (SLTxB) or holotoxin to HeLa cells, and followed its intracellular transport by confocal microscopy. Endocytosed recombinant SLTxB traffics to the ER in the same way as SLTx holotoxin, *via* early endosomes (EE), the *trans*-Golgi network (TGN) and the Golgi stack to the ER [44]. In control cells treated with DMSO or ES_R35_, fluorescently-labeled SLTxB (Cy3-SLTxB) accumulated at a juxta-nuclear position within 40 minutes in those cells that had taken up visible amounts of SLTxB (Figure 4A, Cy3-SLTxB), an accumulation that has been previously shown to occur in the TGN and the Golgi stack [45], [46]. In contrast, in ES_I_ treated cells, SLTxB was not observed in this location until 60 minutes following application, showing a delay in the retrograde trafficking of the toxin B chain. The same qualitative delay was also evident when these experiments were repeated using fluorescently labeled SLTx holotoxin (Figure 4A, Cy2-SLTx).

To define this delay biochemically we used SLTxB-(Sulf)_2_ [46], a recombinant SLTxB chain pentamer whose subunits bear a C-terminal tag that can be radiolabelled with [^35^S]-sulfate upon entry to the *trans*-Golgi cisternae [47]. Relative to control cells treated with DMSO, in ES_I_ -treated cells an initial delay in [^35^S]-labeling of SLTxB-(Sulf)_2_ was observed, indicating delayed entry of SLTxB-(Sulf)_2_ to the *trans*-Golgi cisternae. This was followed by saturated labeling, suggesting a longer residence of the SLTxB-(Sulf)_2_ pentamer in these cisternae upon ES_I_ treatment (Figure 4B, C). On the basis of these results, we conclude that both SLTx entry to the *trans*-Golgi cisternae and subsequent egress from this location are both inhibited by ES_I_ treatment of cells.

### ES_I_ treatment protects cells from diphtheria toxin challenge

**Figure 5:**
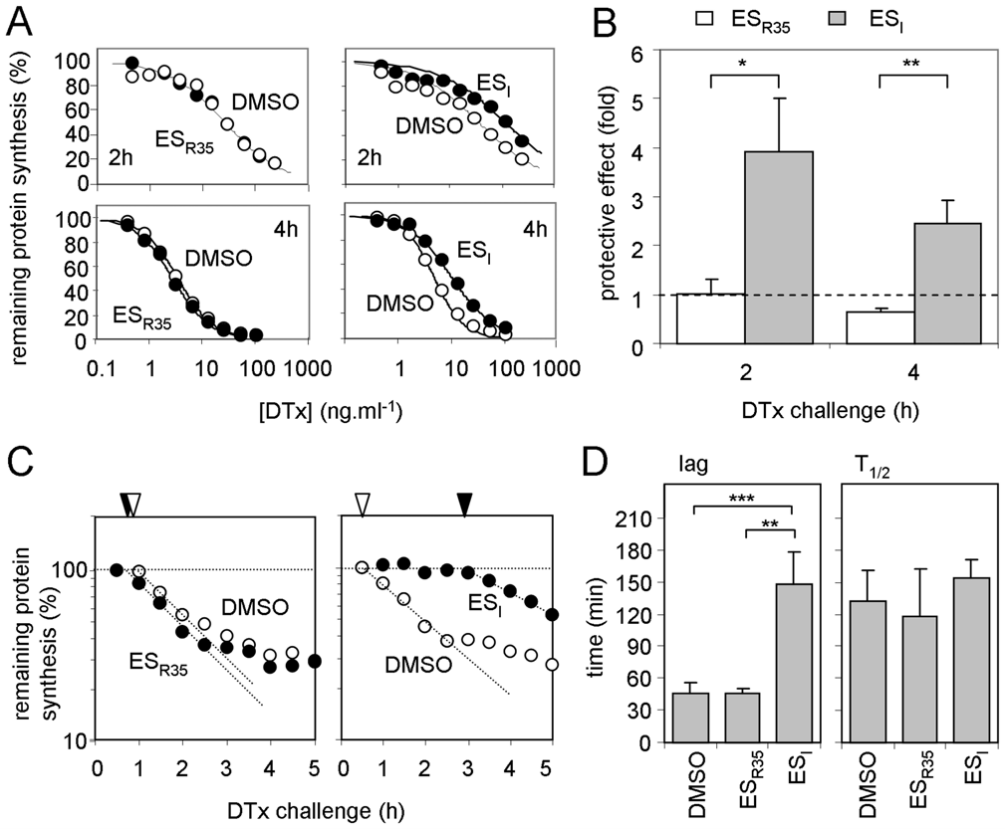
ES_I_ treatment protects cells from DTx challenge. **A.** Typical single cytotoxicity profiles of DMSO, ES_I_ and ES_R35_ treated HeLa cells subsequently challenged with graded doses of DTx for 2 (upper) or 4 h (lower). **B.** Protective effect of the drugs, determined from assays in **A**. n = 3, +/− 1.S.D. Protective effects from ES_I_ and ES_R35_ treated cells were compared using an unpaired t-test (2 h, p = 0.0104, t = 4.555; 4 h, p = .00024, t = 6.858; *, significant: **, very significant). **C.** Typical single assays showing the temporal profile of DTx cytotoxicity to cells treated with DMSO, ES_I_ or ES_R35_, and determination of the lag phase (dotted lines and arrowheads, coded as in Figure 3C). **D.** Lag phases and half-lives of protein synthesis (T_1/2_) were determined. DMSO, n = 6, +/− 1.S.D., ES_I_ and ES_R35_, n = 3, +/− 1 S.D. Values were compared using unpaired t-tests. Lags: DMSO *versus* ES_R35_, no significant difference; DMSO versus ES_I_, p<0.0001, t = 8.810; ES_I_ versus ES_R35_ p = 0.0028, t = 6.573; **, very significant: ***, extremely significant. T_1/2_ values: no significant differences.

SLTx is delivered to the *trans-*Golgi cisternae from an EE compartment via the TGN [1], and therefore the temporal delay in Golgi arrival might reflect reduced rates of trafficking at the endosomal level. In contrast to ER trafficking toxins, following endocytosis diphtheria toxin (DTx) translocates across the endosomal membrane to enter the cytosol as the endosomal pH drops to ≤5.3 (Figure 1) [48]. Strikingly, we found that ES_I_ treatment protected cells from DTx, particularly for shorter intoxication times (Figure 5A, B). In contrast, ES_R35_ had no effect for short intoxications, and may even have had a slight sensitizing effect for longer toxin challenges (Figure 5A, B). The protective effect of ES_I_ again correlated with an increased lag prior to cytosolic delivery of the toxin subunit, with little or no difference measured in the toxin translocation rate (Figure 5C, D).

### ES_I_ treatment delays anterograde trafficking of tsO45VSV-G-GFP from the ER

The retrograde trafficking defects resulting from ES_I_ treatment do not reflect a pleiotropic effect on all aspects of vesicular transport, since the compound has no measureable effect on ricin cytotoxicity (Figure 2B). To investigate further the effects of ES_I_ on vesicular trafficking, we examined anterograde transport in ES_I_-treated cells.

The mutant vesicular stomatitis virus glycoprotein tsO45 VSV-G exhibits temperature sensitive transport from the ER to the cell surface [49] and has become a classical marker for transit through the secretory pathway [50]. At the restrictive temperature (40°C), the protein is misfolded and cycles between the ER, the ER-Golgi intermediate compartment (ERGIC) and the *cis*-Golgi network. However, after a shift to the permissive temperature (32°C), it is released from this cycle and is efficiently trafficked to the cell surface [51]. This system provides an ideal platform from which to investigate the effect of ES_I_ on anterograde transport, independently of any effects of the drug on protein synthesis (Figure 2A) or on membrane integration [38].

**Figure 6:**
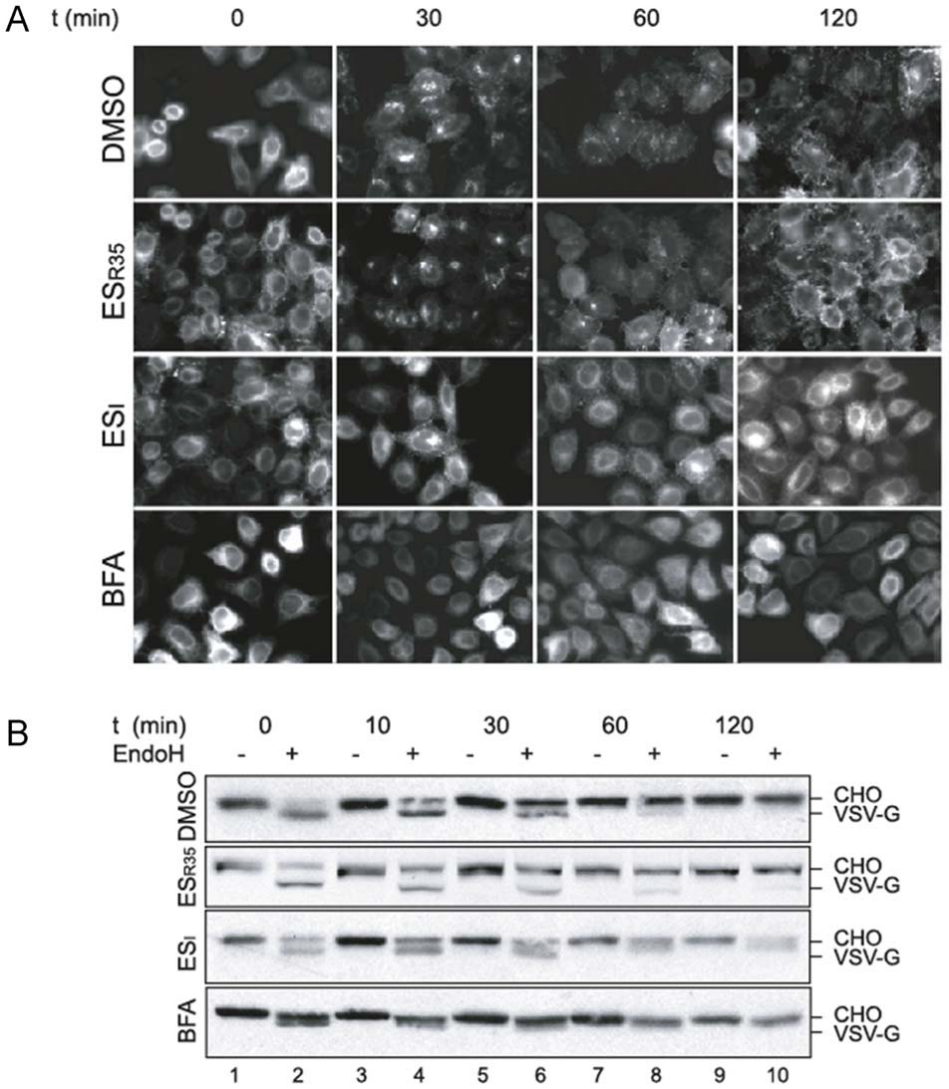
ES_I_ delays anterograde trafficking of tsO45 VSV-G-GFP. **A.** HeLa cells infected with adenovirus encoding tsO45 VSV-G-GFP were grown overnight at 40°C, then pre-treated with DMSO, with 8 µM ES_R35_, ES_I_, or with 5 µg.ml^-1^ Brefeldin A (BFA) for 1 h. Cells were then shifted to 32°C, and incubated for the time indicated in the continued presence of the compounds, prior to fixation and visualisation by fluorescence microscopy. **B.** Lysates of HeLa cells treated as in **A** were incubated with Endoglycosidase H (EndoH, +) or mock treated (−), then analysed by SDS-PAGE followed by immunoblotting using anti-VSVG antibody and alkaline phosphatase conjugated secondary antibody. The position of *N*-glycosylated (CHO) and non-glycosylated VSV-G are indicated.

Cells were infected with adenovirus encoding a GFP-tagged tsO45 VSV-G (tsO45 VSV-G –GFP) and grown overnight at 40°C. Upon a temperature shift from 40°C to 32°C, tsO45 VSV-G-GFP was released from the early secretory pathway, accumulating in a peri-nuclear region by ∼30 min and appearing primarily at the cell surface after 1 to 2 hours in cells incubated with DMSO or ES_R35_ (Figure 6A, DMSO and ES_R35_ panels). In contrast, when cells were treated with the fungal metabolite Brefeldin A (BFA), which blocks secretion by stimulating Golgi and ER membrane fusion [52], [53], [54], tsO45 VSV-G-GFP remained within the fused ER-Golgi compartment following the temperature shift and did not reach the cell surface even after 2 hours (Figure 6A, BFA panels). In most ES_I_ treated cells, the bulk of the tsO45 VSV-G-GFP protein remained at an intracellular location even after 2 hours, although some degree of cell surface labeling was apparent at both 1 and 2 hours (Figure 6A, ES_I_ panels).

To underpin this microscopy-based analysis of VSV-G trafficking, we also examined Golgi-dependent alterations in the *N*-linked glycans of the VSV-G protein to investigate its progress through the secretory pathway. During passage through the Golgi, the *N*-linked glycans of VSV-G are modified such that they acquire resistance to in vitro removal by the enzyme endoglycoside H (EndoH) [55]. In lysates of HeLa cells expressing tsO45 VSV-G, ES_I_ treatment delayed the conversion of the glycoprotein into an EndoH-resistant form, such that even after 2 hours a population of EndoH-sensitive glycoprotein was still present (Figure 6B, compare ES_I_ with DMSO and ES_R35_). In the case of BFA, we noted a more rapid acquisition of EndoH resistance (Figure 6B, BFA) consistent with the entry of Golgi α-mannosidase II (whose activity converts *N*-glycans from EndoH sensitivity to resistance) into the ER upon BFA treatment [56]. Taken together, these data indicate that the anterograde trafficking of VSV-G through the secretory pathway, and its appearance at the cell surface, are delayed by ES_I_ treatment.

Finally, we examined the effects of ES_I_ on the morphology of organelles used by intracellular trafficking toxins. Typically, long drug pretreatments (8–48 h) have been used in experiments that demonstrate efficacy of ES_I_­ in retarding the dislocation of an ERAD substrate [34], inhibition of p97-associated functions [35], and linkage of ER stress with activation of the ATF3 and ATF4 transcription factors [57]. However, even with exposures as short as 1 hour, ES_I_ exhibits biological activity, with clear effects on protein translocation into the ER [38]. Immunofluorescence microscopy revealed that 8 h treatment with ES_I_ resulted in extensive vacuolation of the ER consistent with pathological effects resulting from ER stress [58]. In contrast, there were no obvious effects on lysosomal and Golgi morphology (Figure S1). Importantly, the effects of ES_I_ treatment on the cytotoxicity of ER-dislocating toxins shown here were performed at shorter time intervals, specifically before any overt changes in cellular morphology were apparent (data not shown).

## Discussion

We show that ES_I_ interferes with both anterograde and retrograde transport of vesicular cargoes. Although no obvious morphological changes to Golgi structure were seen even after extended (8 hour) treatments of HeLa cells with ES_I_, we detected a clear delay in the trafficking of VSV-G protein through the Golgi and its subsequent appearance at the cell surface. The nature of the VSV-G trafficking assay allows us to ascribe this as an effect on vesicular transport, and this effect is consistent with our previous observation that ES_I_ treatment almost completely inhibits the secretion of newly synthesized proteins from HepG2 cells [38]. When taken together with the data presented here, we conclude that the strong ES_I_ dependent inhibition of protein secretion that we previously observed is most likely due to multiple effects, with the drug reducing rates of protein synthesis (this study), affecting Sec61-mediated translocation across the ER membrane [38] and delaying subsequent vesicular transport to the cell surface (this study).

ES_I_ treatment was also found to inhibit the retrograde transport of DTx to low pH endosomes. The initial stages of DTx uptake rely on the endocytic pathway of the host cell, and our data indicate that the effects of ES_I_ with respect to DTx are at this early stage of the process and not at the later membrane translocation step (Figure 1).

In addition, ES_I_ treatment inhibits the retrograde transport of SLTx to and through the Golgi stack. Strikingly however, there was no measurable affect on the cytotoxicity of ricin, which, like SLTx, is thought to traffic via EE, the TGN and Golgi to the ER (Figure 1). Despite the superficial similarities in retrograde trafficking of ricin and SLTx, there are a number of characterized differences, particularly during transit through the Golgi. Hence, SLTx traverses the Golgi in a Rab6A'-dependent manner [59], [60], [61], whilst ricin traffics through the Golgi in a Rab6A'-independent manner [62]. Other differences include the strong inhibition of SLTx trafficking but weak inhibition of ricin trafficking by knockdown of syntaxin 5 [63] and the different requirements for sorting nexins at the endosome-TGN interface [64], [65], [66], [67]. Thus ES_I_ does not act as a general inhibitor of vesicular trafficking, since at least one retrograde pathway is unresponsive to treatment (see Figure 1). The substrate specificity of ES_I_ inhibition further underlines that there are multiple routes or mechanisms by which exogenous toxins can reach the ER by retrograde transport [68], [1].

ES_I_ is unlikely to inhibit toxin action directly, since DTx and SLTxA1 have different targets and mechanisms of action (elongation factor-2 modification and ribosome modification, respectively), and the rate of intoxication after the initial lag phase is unaltered for both of these toxins. We cannot formally exclude the possibility that one or more of the currently suggested modes of action of ES_I_, namely interference with early dislocation [34], p97-associated [12], [35] or ER translocation processes [38], results in the selective inhibition of specific intracellular trafficking routes. However, we found no effect of ES_I_ treatment upon the kinetics of dislocation of any of the toxins that we investigated in this study. In the case of RTA and SLTxA, this suggests that the ER-cytosol dislocation of both of these toxins does not involve the early ES_I_ inhibited step identified by Fiebiger and colleagues [34], or either of the two proposed targets of ESI: p97 and the Sec61 translocon [12], [35], [38]. A recent report noted ES_I_-induced cross-links between PDI and an ERAD substrate [39]. PDI is thought to be responsible for the reductive cleavage of ricin, releasing RTA chain from its RTB partner in the ER lumen, a necessary prerequisite for the ER dislocation of the A chain [2], [3]. Since ES_I_ had no effect on the cytotoxicity of ricin, it seems unlikely that it induced cross-links between RTA and PDI over the short time scales used for cytotoxicity measurements. However, it should be noted that the inhibitory effect of ES_I_ when applied to intact cells is incomplete [38]. Thus it is also conceivable that these previously defined steps or components are perturbed by ES_I_, but that they are not rate-limiting for toxin dislocation across the ER membrane.

ES_I_ has a bi-modular structure, with a comparatively hydrophobic portion that acts as a membrane binding domain [36] and a 5-nitrofuran containing group that is required for its effects on protein secretion and ER translocation [38], as well as p97 function [36]. ES_R35_, which lacks the nitrofuran group of ES_I_, was ineffective in all of our assays. A number of studies have shown that 5-nitrofuran containing compounds cause diverse effects in biological systems including the inhibition of *Salmonella* growth [69] and topisomerase function [70] and the induction of oxidative stress [71]. Given these wide-ranging effects and the capacity of nitrofurans to undergo *in vivo* modifications [72], we conclude that the most likely explanation of our data is that ES_I_ acts on one or more currently unidentified components that function during vesicular transport. The siRNA-mediated perturbation of signal recognition particle-dependent protein targeting to the Sec61 translocon has been shown to result in selective defects in post-ER membrane trafficking [73]. Hence the ES_I_ dependent inhibition of Sec61 mediated translocation [38] could potentially contribute to the effects that we report here. However, as we saw an inhibitory effect within comparatively short timescales we believe it is more likely that components involved in vesicular trafficking are affected directly by ES_I_, rather than being depleted as a consequence of any reduction in their synthesis or Sec61 mediated translocation. Given the inhibitory effect of ES_I_ upon p97-associated de-ubiquitylation [35], [36], [37], one possibility is that the compound may also impact one or more of the deubiquitinases implicated in endocytosis [74].

In the original study by Fiebiger et al., [34] the authors found that ES_I_ inhibits both the human cytomegalovirus protein US11-dependent degradation of MHC class I molecules, and the US11-independent degradation of TCRα, a well-defined model for a cellular ERAD substrate. The authors concluded that ES_I_ inhibits one or more stages just prior to, or concomitant with, dislocation from the ER. Given that a number of ERAD substrates appear to cycle between the ER and Golgi as a pre-requisite for subsequent dislocation [75], [76], [77], [21], and evidence of cross-talk between the secretory and ER degradative pathways [78], our new data raise the possibility that a perturbation of vesicular transport by ES_I_ may also contribute to the drug's inhibition of ER associated protein degradation.

## Materials and Methods

### Cell culture

HeLa cells that reliably express the SLTx receptor Gb3 [47] were used for all the cytotoxicity and SLTx trafficking experiments. HeLa cells used for the VSV-G assays and the data shown in Figure S1 were obtained from ATCC (CCL-2™). Cells were maintained in Dulbecco's modified Eagle's medium (DMEM) supplemented with 10% fetal calf serum (DMEM/FCS), and grown at 37°C in a 5% CO_2_ atmosphere.

### Cytotoxicity experiments

Cytotoxicity experiments were performed on cells seeded at 2×10^4^ cells per well of a 96-well plate after overnight growth. Growth medium was replaced by DMEM/FCS containing drug (4 µM, from a stock dissolved in DMSO) or vehicle for 1 h. After this pretreatment, the medium was replaced by serial dilutions of toxin in DMEM/FCS containing either drug or vehicle as appropriate, and after incubation (see Figures for toxin incubation periods), the cells were rinsed in phosphate buffered saline, pH 7.4 (PBS) and incubated for 15 minutes in PBS containing 1 µCi.ml-1 [^35^S]-methionine/cysteine (Perkin Elmer). Wells were then rinsed in PBS, and washed 5 times with cold 5% trichloroacetic acid. After addition of 200 µl Optiphase supermix scintillant (Perkin Elmer) to each drained well, and incubation at room temperature (∼2 h), radioactivity incorporated into acid-precipitable material was determined by scintillation counting in a 1450 MicroBeta Trilux scintillation counter (Perkin Elmer). In all cases, toxin titration experiments for drug and vehicle were performed at the same time on the same 96-well plate, to remove any differences between plates. Data were normalized to coeval controls (nontoxin treated cells pretreated and treated with drug or vehicle treated as appropriate) and represent remaining protein synthesis (%). Concentrations (IC_50_) of toxin that reduced protein synthesis ability to 50% were determined using a median effect plot [79], and protective effects were calculated as the ratio of IC_50_ drug:IC_50_ vehicle. For time course cytotoxicity experiments, cells were treated with a fixed concentration (1 µg.ml^−1^) of toxin for increasing times (see Figures for details), but were otherwise treated as above.

### Microscopy

HeLa cells seeded at 2×10^5^ cells/well in 1 ml complete DMEM/FCS were grown overnight on methanol-sterilised coverslips in 12-well plates. They were then pre-treated with 0.1% DMSO, 4 µM ES_I_ or 4 µM ES_R35_ for 1 hour and then incubated in 250 µl ice-cold PBS (containing DMSO, ES_I_ or ES_R35_ as appropriate) and incubated with either Cy2-SLTx or Cy3-SLTxB for 30 minutes at 4°C. The PBS was then replaced with 1 ml of complete DMEM/FCS and incubated at 37°C and cells were fixed using 4% paraformaldehyde (PFA) as part of a time-course. The final time-point was fixed with 4% PFA at 37°C for 20 minutes. Following fixing, PFA was quenched using 30 mM glycine for 5 minutes and washed three times in PBS before mounting using Vectashield mounting medium containing DAPI (Vector Labs, Burlinghame, CA).

HeLa cells exposed to both Cy2-SLTx and Cy3-SLTxB were imaged sequentially using Leica SP5 confocal microscopy. Cells treated with Cy3-SLTx were imaged using the DPSS 561 laser with an emission bandwidth between 566 nm to 790 nm (red) and the 405 diode ultraviolet (UV) laser with an emission bandwidth between 381 nm to 477 nm to image the nuclei (blue). The argon laser (at 20%) was used to collect differential interface contrast (DIC) images using the 488 nm laser. Cells treated with Cy2-SLTxB were imaged using the argon laser (at 20%) utilizing the 488 nm laser with an emission bandwidth between 497 nm to 638 nm (green), also used to collect DIC images. Again the 405 diode UV laser with an emission bandwidth between 414 nm to 488 nm was used to image the nuclei (blue).

### GFP-tagged VSV-G trafficking

HeLa cells seeded at 3×10^5^ cells/well in 2 ml complete DMEM/FCS were grown overnight on methanol-sterilised coverslips in 6-well plates and infected with adenovirus. Encoding a GFP-tagged tsO45 VSV-G protein as described by Chui et al [80]. Post-infection, the medium was replaced with fresh DMEM/FCS and the cells were kept at 40°C overnight. The following day, cells were pretreated for 1 h at 40°C in fresh carbonate free medium containing DMSO (vehicle control), 8 µM ES_I_, 8 µM ES_R35_ or 5 µg.ml^−1^ Brefeldin A as appropriate. The cells were then shifted to the permissive temperature (32°C) and were fixed as above after various lengths of incubation (see appropriate Figure legends). Cells were mounted in Mowiol and imaged using an Olympus BX60 upright microscope and Metamorph software (Universal Imaging Corp.). Alternatively cells wre harvested in Triton IP buffer (10 mM Tris-HCl pH 7.6, 140 mM NaCl, I mM EDTA, 1% Triton x-100) and after a brief spin, the post-nuclear supernatant. was split into two aliquots. One aliquot was incubated overnight with 6670 U.ml^−1^ of Endoglycosidase H as directed by the supplier (New England Biolabs). Samples were analysed by SDS-PAGE and immunoblotting with mouse anti-VSV-G antibody followed by alkaline phospatase conjugated anti-mouse-antibodiy (both from Sigma) and visualized by development with BCIP/NBT colour development substrate (Promega).

### SLTx and SLTxB labeling with Cy dyes

Purified SLTx and SLTxB were labeled according to the manufacturer's instructions (GE Healthcare).

### SLTxB sulfation assay

Cells were seeded at 10^6^ per well in six-well plates, grown overnight and washed twice in sulfate-free DMEM (Gibco) lacking FCS. They were then incubated for 3 h, 37°C in sulfate-free medium lacking FCS in the presence of 200 µCi.ml^−1^ [^35^S]-SO_4_. During the last hour of this period, cells were treated with drug or vehicle as appropriate. SLTxB (1 µl, 1 mg.ml^−1^) was added to wells and incubation continued for a time-course (see Figures and legends). Cells were then washed twice with cold PBS and scraped into 1 ml of lysis buffer (50 mM Tris-HCl pH 7.5, 1% Triton X-100) containing protease inhibitor cocktail (Roche). Cell debris was removed by brief centrifugation (1 minute, 14,000 rpm, in a microfuge) and SLTxB chain was immunoprecipitated from the soluble fraction with sheep anti SLTxB and proteinA-Sepharose at 4°C overnight and washed as previously described [3]. Sulfated SLTxB was identified by autoradiography after SDS-PAGE [81] of the immunoprecipitated material.

### Casein degradation assays

Human erythrocyte 26S proteasomes (10 nM, Enzo Life Sciences) were pre-incubated in 20 mM Hepes pH 7.6, 4 mM ATP, 10 mM MgCl_2_, 1 mM DTT containing 20 µM clasto-Lactacystin β-lactone (Merck) or its vehicle DMSO for 30 minutes at 24°C before addition of 5 µM β-casein (Sigma-Aldritch). After incubation (4 h, 37°C), the samples were subjected to SDS-PAGE and immunoblotting. Casein was revealed by sequential incubations of the blot with sheep anti-casein antibodies (Thermo Scientific) and donkey anti-sheep antibodies conjugated to alkaline phosphatase (Sigma Aldrich) followed by development with BCIP/NBT colour development substrate (Promega).

## Supporting Information

Figure S1 **Effect of ES_I_ on subcellular morphology.** HeLa cells were treated with 8 µM ES_R35_ or ES_I_, or left untreated (UT) for 8 h, then fixed with methanol. Different subcellular compartments were visualised by labelling with anti-PDI (ER), anti-LAMP1 (lysosomes) or anti-Golgin 84 (Golgi appuratus), followed by fluorescently labelled secondary antibodies.
(TIF)

## References

[1] Spooner RA, Smith DC, Easton AJ, Roberts LM, Lord JM (2006). Retrograde transport pathways utilised by viruses and protein toxins.. Virol J.

[2] Bellisola G, Fracasso G, Ippoliti R, Menestrina G, Rosen A (2004). Reductive activation of ricin and ricin A-chain immunotoxins by protein disulfide isomerase and thioredoxin reductase.. Biochem Pharmacol.

[3] Spooner RA, Watson PD, Marsden CJ, Smith DC, Moore KA (2004). Protein disulphide-isomerase reduces ricin to its A and B chains in the endoplasmic reticulum.. Biochem J.

[4] Mayerhofer PU, Cook JP, Wahlman J, Pinheiro TT, Moore KA (2009). Ricin A chain insertion into endoplasmic reticulum membranes is triggered by a temperature increase to 37°C.. J Biol Chem.

[5] Ye Y, Shibata Y, Yun C, Ron D, Rapoport TA (2004). A membrane protein complex mediates retro-translocation from the ER lumen into the cytosol.. Nature.

[6] Sun F, Zhang R, Gong X, Geng X, Drain PF (2006). Derlin-1 promotes the efficient degradation of the cystic fibrosis transmembrane conductance regulator (CFTR) and CFTR folding mutants.. J Biol Chem.

[7] Tsai YC, Weissman AM (2010). The Unfolded Protein Response, Degradation from Endoplasmic Reticulum and Cancer.. Genes Cancer.

[8] Rabinovich E, Kerem A, Frohlich KU, Diamant N, Bar-Nun S (2002). AAA-ATPase p97/Cdc48p, a cytosolic chaperone required for endoplasmic reticulum-associated protein degradation.. Mol Cell Biol.

[9] Elkabetz Y, Shapira I, Rabinovich E, Bar-Nun S (2004). Distinct steps in dislocation of luminal endoplasmic reticulum-associated degradation substrates: roles of endoplamic reticulum-bound p97/Cdc48p and proteasome.. J Biol Chem.

[10] Ye Y, Meyer HH, Rapoport TA (2001). The AAA ATPase Cdc48/p97 and its partners transport proteins from the ER into the cytosol.. Nature.

[11] Stirling CJ, Lord JM (2006). Quality control: linking retrotranslocation and degradation.. Curr Biol.

[12] Wang Q, Li L, Ye Y (2006). Regulation of retrotranslocation by p97-associated deubiquitinating enzyme ataxin-3.. J Cell Biol.

[13] Zhong X, Pittman RN (2006). Ataxin-3 binds VCP/p97 and regulates retrotranslocation of ERAD substrates.. Hum Mol Genet.

[14] Medicherla B, Kostova Z, Schaefer A, Wolf DH (2004). A genomic screen identifies Dsk2p and Rad23p as essential components of ER-associated degradation.. EMBO Rep.

[15] Wesche J, Rapak A, Olsnes S (1999). Dependence of ricin toxicity on translocation of the toxin A-chain from the endoplasmic reticulum to the cytosol.. J Biol Chem.

[16] Willer M, Forte GM, Stirling CJ (2008). Sec61p is required for ERAD-L: genetic dissection of the translocation and ERAD-L functions of Sec61P using novel derivatives of CPY.. J Biol Chem.

[17] Slominska-Wojewodzka M, Gregers TF, Walchli S, Sandvig K (2006). EDEM is involved in retrotranslocation of ricin from the endoplasmic reticulum to the cytosol.. Mol Biol Cell.

[18] Redmann V, Oresic K, Tortorella LL, Cook JP, Lord M (2011). Dislocation of ricin toxin A chains in human cells utilizes selective cellular factors.. J Biol Chem.

[19] Spooner RA, Hart PJ, Cook JP, Pietroni P, Rogon C (2008). Cytosolic chaperones influence the fate of a toxin dislocated from the endoplasmic reticulum.. Proc Natl Acad Sci U S A.

[20] Endo Y, Tsurugi K (1987). RNA N-glycosidase activity of ricin A-chain. Mechanism of action of the toxic lectin ricin on eukaryotic ribosomes.. J Biol Chem.

[21] Li S, Spooner RA, Allen SC, Guise CP, Ladds G (2010). Folding-competent and folding-defective forms of ricin A chain have different fates after retrotranslocation from the endoplasmic reticulum.. Mol Biol Cell.

[22] Endo Y, Tsurugi K, Yutsudo T, Takeda Y, Ogasawara T (1988). Site of action of a Vero toxin (VT2) from Escherichia coli O157:H7 and of Shiga toxin on eukaryotic ribosomes. RNA N-glycosidase activity of the toxins.. Eur J Biochem.

[23] Yu M, Haslam DB (2005). Shiga toxin is transported from the endoplasmic reticulum following interaction with the luminal chaperone HEDJ/ERdj3.. Infect Immun.

[24] Falguieres T, Johannes L (2006). Shiga toxin B-subunit binds to the chaperone BiP and the nucleolar protein B23.. Biol Cell.

[25] Williams DB, Watts TH (1995). Molecular chaperones in antigen presentation.. Curr Opin Immunol.

[26] Cresswell P (2000). Intracellular surveillance: controlling the assembly of MHC class I-peptide complexes.. Traffic.

[27] Tortorella D, Gewurz BE, Furman MH, Schust DJ, Ploegh HL (2000). Viral subversion of the immune system.. Annu Rev Immunol.

[28] Wiertz EJ, Jones TR, Sun L, Bogyo M, Geuze HJ (1996). The human cytomegalovirus US11 gene product dislocates MHC class I heavy chains from the endoplasmic reticulum to the cytosol.. Cell.

[29] Mueller B, Lilley BN, Ploegh HL (2006). SEL1L, the homologue of yeast Hrd3p, is involved in protein dislocation from the mammalian ER.. J Cell Biol.

[30] Mueller B, Klemm EJ, Spooner E, Claessen JH, Ploegh HL (2008). SEL1L nucleates a protein complex required for dislocation of misfolded glycoproteins.. Proc Natl Acad Sci U S A.

[31] Baker BM, Tortorella D (2007). Dislocation of an endoplasmic reticulum membrane glycoprotein involves the formation of partially dislocated ubiquitinated polypeptides.. J Biol Chem.

[32] Schulze A, Standera S, Buerger E, Kikkert M, van Voorden S (2005). The ubiquitin-domain protein HERP forms a complex with components of the endoplasmic reticulum associated degradation pathway.. J Mol Biol.

[33] Burr ML, Cano F, Svobodova S, Boyle LH, Boname JM (2011). HRD1 and UBE2J1 target misfolded MHC class I heavy chains for endoplasmic reticulum-associated degradation.. Proc Natl Acad Sci U S A.

[34] Fiebiger E, Hirsch C, Vyas JM, Gordon E, Ploegh HL (2004). Dissection of the dislocation pathway for type I membrane proteins with a new small molecule inhibitor, eeyarestatin.. Mol Biol Cell.

[35] Wang Q, Li L, Ye Y (2008). Inhibition of p97-dependent protein degradation by Eeyarestatin I.. J Biol Chem.

[36] Wang Q, Shinkre BA, Lee JG, Weniger MA, Liu Y (2010). The ERAD inhibitor Eeyarestatin I is a bifunctional compound with a membrane-binding domain and a p97/VCP inhibitory group.. PLoS One.

[37] Dolan BP, Li L, Veltri CA, Ireland CM, Bennink JR (2011). Distinct pathways generate peptides from defective ribosomal products for CD8+ T cell immunosurveillance.. J Immunol.

[38] Cross BC, McKibbin C, Callan AC, Roboti P, Piacenti M (2009). Eeyarestatin I inhibits Sec61-mediated protein translocation at the endoplasmic reticulum.. J Cell Sci.

[39] Ernst R, Claessen JHL, Mueller B, Sanyal S, Spooner E (2011). Enzymatic blockade of the ubiquitin-proteasome pathway.. PLoS Biol.

[40] Craiu A, Gaczynska M, Akopian T, Gramm CF, Fenteany G (1997). Lactacystin and clasto-lactacystin beta-lactone modify multiple proteasome beta-subunits and inhibit intracellular protein degradation and major histocompatibility complex class I antigen presentation.. J Biol Chem.

[41] Tam PJ, Lingwood CA (2007). Membrane cytosolic translocation of verotoxin A1 subunit in target cells.. Microbiology.

[42] Hudson TH, Neville DM (1987). Temporal separation of protein toxin translocation from processing events.. J Biol Chem.

[43] Neville DM, Youle RJ (1982). Monoclonal antibody-ricin or ricin A chain hybrids: kinetic analysis of cell killing for tumor therapy.. Immunol Rev.

[44] Sandvig K, Ryd M, Garred O, Schweda E, Holm PK (1994). Retrograde transport from the Golgi complex to the ER of both Shiga toxin and the nontoxic Shiga B-fragment is regulated by butyric acid and cAMP.. J Cell Biol.

[45] Sandvig K, Garred O, Prydz K, Kozlov JV, Hansen SH (1992). Retrograde transport of endocytosed Shiga toxin to the endoplasmic reticulum.. Nature.

[46] Mallard F, Antony C, Tenza D, Salamero J, Goud B (1998). Direct pathway from early/recycling endosomes to the Golgi apparatus revealed through the study of shiga toxin B-fragment transport.. J Cell Biol.

[47] Spooner RA, Watson P, Smith DC, Boal F, Amessou M (2008). The secretion inhibitor Exo2 perturbs trafficking of Shiga toxin between endosomes and the trans-Golgi network.. Biochem J.

[48] Blewitt MG, Chung LA, London E (1985). Effect of pH on the conformation of diphtheria toxin and its implications for membrane penetration.. Biochemistry.

[49] Gallione CJ, Rose JK (1985). A single amino acid substitution in a hydrophobic domain causes temperature-sensitive cell-surface transport of a mutant viral glycoprotein.. J Virol.

[50] Presley JF, Cole NB, Schroer TA, Hirschberg K, Zaal KJ (1997). ER-to-Golgi transport visualized in living cells.. Nature.

[51] Hammond C, Helenius A (1994). Quality control in the secretory pathway: retention of a misfolded viral membrane glycoprotein involves cycling between the ER, intermediate compartment, and Golgi apparatus.. J Cell Biol.

[52] Donaldson JG, Lippincott-Schwartz J, Bloom GS, Kreis TE, Klausner RD (1990). Dissociation of a 110-kD peripheral membrane protein from the Golgi apparatus is an early event in brefeldin A action.. J Cell Biol.

[53] Orci L, Tagaya M, Amherdt M, Perrelet A, Donaldson JG (1991). Brefeldin A, a drug that blocks secretion, prevents the assembly of non-clathrin-coated buds on Golgi cisternae.. Cell.

[54] Lippincott-Schwartz J, Yuan LC, Bonifacino JS, Klausner RD (1989). Rapid redistribution of Golgi proteins into the ER in cells treated with brefeldin A: evidence for membrane cycling from Golgi to ER.. Cell.

[55] Chen SS, Huang AS (1986). Further characterization of the vesicular stomatitis virus temperature-sensitive O45 mutant: intracellular conversion of the glycoprotein to a soluble form.. J Virol.

[56] Scheel J, Pepperkok R, Lowe M, Griffiths G, Kreis TE (1997). Dissociation of coatomer from membranes is required for brefeldin A-induced transfer of Golgi enzymes to the endoplasmic reticulum.. J Cell Biol.

[57] Wang Q, Mora-Jensen H, Weniger MA, Perez-Galan P, Wolford C (2009). ERAD inhibitors integrate ER stress with an epigenetic mechanism to activate BH3-only protein NOXA in cancer cells.. Proc Natl Acad Sci U S A.

[58] Kar R, Singha PK, Venkatachalam MA, Saikumar P (2009). A novel role for MAP1 LC3 in nonautophagic cytoplasmic vacuolation death of cancer cells.. Oncogene.

[59] White J, Johannes L, Mallard F, Girod A, Grill S (1999). Rab6 coordinates a novel Golgi to ER retrograde transport pathway in live cells.. J Cell Biol.

[60] Monier S, Jollivet F, Janoueix-Lerosey I, Johannes L, Goud B (2002). Characterization of novel Rab6-interacting proteins involved in endosome-to-TGN transport.. Traffic.

[61] Del Nery E, Miserey-Lenkei S, Falguieres T, Nizak C, Johannes L (2006). Rab6A and Rab6A' GTPases play non-overlapping roles in membrane trafficking.. Traffic.

[62] Chen A, AbuJarour RJ, Draper RK (2003). Evidence that the transport of ricin to the cytoplasm is independent of both Rab6A and COPI.. J Cell Sci.

[63] Amessou M, Fradagrada A, Falguieres T, Lord JM, Smith DC (2007). Syntaxin 16 and syntaxin 5 are required for efficient retrograde transport of several exogenous and endogenous cargo proteins.. J Cell Sci.

[64] Skönland SS, Walchli S, Utskarpen A, Wandinger-Ness A, Sandvig K (2007). Phosphoinositide-regulated retrograde transport of ricin: crosstalk between hVps34 and sorting nexins.. Traffic.

[65] Dyve AB, Bergan J, Utskarpen A, Sandvig K (2009). Sorting nexin 8 regulates endosome-to-Golgi transport.. Biochem Biophys Res Commun.

[66] Bujny MV, Popoff V, Johannes L, Cullen PJ (2007). The retromer component sorting nexin-1 is required for efficient retrograde transport of Shiga toxin from early endosome to the trans Golgi network.. J Cell Sci.

[67] Popoff V, Mardones GA, Bai SK, Chambon V, Tenza D (2009). Analysis of articulation between clathrin and retromer in retrograde sorting on early endosomes.. Traffic.

[68] Smith DC, Spooner RA, Watson PD, Murray JL, Hodge TW (2006). Internalized Pseudomonas exotoxin A can exploit multiple pathways to reach the endoplasmic reticulum.. Traffic.

[69] Chadfield MS, Hinton MH (2004). In vitro activity of nitrofuran derivatives on growth and morphology of Salmonella enterica serotype Enteritidis.. J Appl Microbiol.

[70] Polycarpou-Schwarz M, Muller K, Denger S, Riddell A, Lewis J (2007). Thanatop: a novel 5-nitrofuran that is a highly active, cell-permeable inhibitor of topoisomerase II.. Cancer Res.

[71] Maya JD, Bollo S, Nunez-Vergara LJ, Squella JA, Repetto Y (2003). Trypanosoma cruzi: effect and mode of action of nitroimidazole and nitrofuran derivatives.. Biochem Pharmacol.

[72] Letelier ME, Izquierdo P, Godoy L, Lepe AM, Faundez M (2004). Liver microsomal biotransformation of nitro-aryl drugs: mechanism for potential oxidative stress induction.. J Appl Toxicol.

[73] Lakkaraju AK, Luyet PP, Parone P, Falguieres T, Strub K (2007). Inefficient targeting to the endoplasmic reticulum by the signal recognition particle elicits selective defects in post-ER membrane trafficking.. Exp Cell Res.

[74] Clague MJ, Urbe S (2006). Endocytosis: the DUB version.. Trends Cell Biol.

[75] Wakana Y, Takai S, Nakajima K, Tani K, Yamamoto A (2008). Bap31 is an itinerant protein that moves between the peripheral endoplasmic reticulum (ER) and a juxtanuclear compartment related to ER-associated Degradation.. Mol Biol Cell.

[76] Hosokawa N, You Z, Tremblay LO, Nagata K, Herscovics A (2007). Stimulation of ERAD of misfolded null Hong Kong alpha1-antitrypsin by Golgi alpha1,2-mannosidases.. Biochem Biophys Res Commun.

[77] Vashist S, Kim W, Belden WJ, Spear ED, Barlowe C (2001). Distinct retrieval and retention mechanisms are required for the quality control of endoplasmic reticulum protein folding.. J Cell Biol.

[78] Fu L, Sztul E (2003). Traffic-independent function of the Sar1p/COPII machinery in proteasomal sorting of the cystic fibrosis transmembrane conductance regulator.. J Cell Biol.

[79] Aghi M, Kramm CM, Chou TC, Breakefield XO, Chiocca EA (1998). Synergistic anticancer effects of ganciclovir/thymidine kinase and 5-fluorocytosine/cytosine deaminase gene therapies.. J Natl Cancer Inst.

[80] Chiu CF, Ghanekar Y, Frost L, Diao A, Morrison D (2008). ZFPL1, a novel ring finger protein required for cis-Golgi integrity and efficient ER-to-Golgi transport.. Embo J.

[81] Schagger H, von Jagow G (1987). Tricine-sodium dodecyl sulfate-polyacrylamide gel electrophoresis for the separation of proteins in the range from 1 to 100 kDa.. Anal Biochem.

