# Supplementary figures and images for: Eeyarestatin 1 Interferes with Both Retrograde and Anterograde Intracellular Trafficking Pathways
Source: PLoS One. 2011 Jul 25;6(7):e22713. doi: 10.1371/journal.pone.0022713 (PMC3143184; doi:10.1371/journal.pone.0022713)

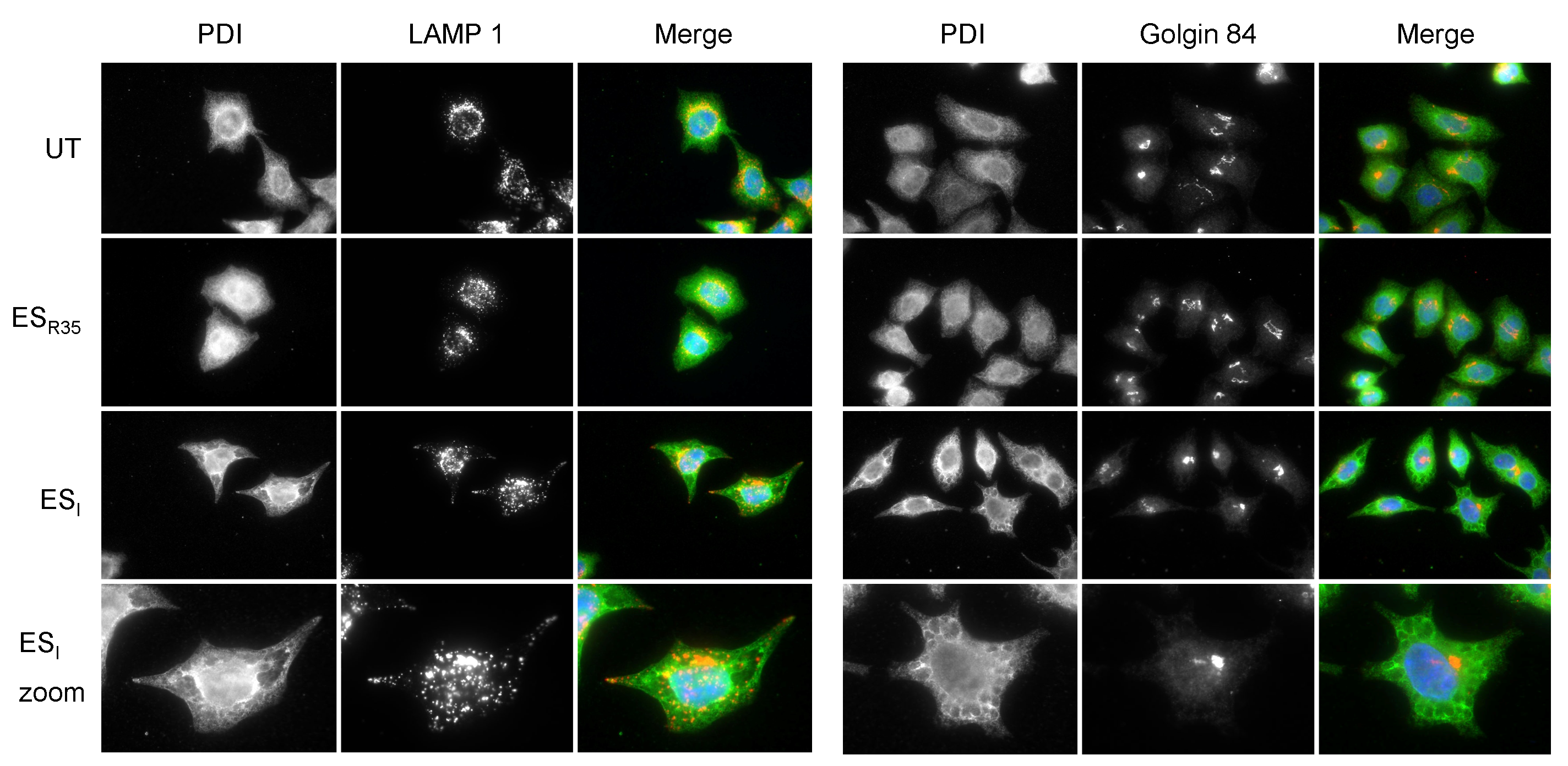

Supplement: Figure S1 — Effect of ESI on subcellular morphology. HeLa cells were treated with 8 µM ESR35 or ESI, or left untreated (UT) for 8 h, then fixed with methanol. Different subcellular compartments were visualised by labelling with anti-PDI (ER), anti-LAMP1 (lysosomes) or anti-Golgin 84 (Golgi appuratus), followed by fluorescently labelled secondary antibodies. (TIF) [file pone.0022713.s001.tif]
